# Intermittent Losartan Administration Triggers Cardiac Post-Conditioning in Isolated Rat Hearts: Role of BK2 Receptors

**DOI:** 10.1371/journal.pone.0088542

**Published:** 2014-02-10

**Authors:** Luca Sgarra, Valentina Leo, Francesco Addabbo, Dominga Iacobazzi, Maria Rosaria Carratù, Monica Montagnani, Maria Assunta Potenza

**Affiliations:** Department of Biomedical Sciences and Human Oncology, University of Bari “Aldo Moro,” Bari, Italy; Facultad de Medicina, Universidad Autonoma Madrid, Spain

## Abstract

**Introduction:**

The angiotensin (Ang) and bradykinin (BK) tissue-system plays a pivotal role in post-conditioning, but the efficacy of angiotensin type 1 receptor (AT1R) blockers (ARBs) in post-ischemic strategies is still under investigation. We evaluated functional and morphological outcomes, together with activation of cytosolic RISK pathway kinases, in rat hearts subjected to losartan (LOS) or irbesartan (IRB) post-ischemic administration.

**Methods:**

Isolated rat hearts underwent 30 min ischemia and 120 min reperfusion. Post-conditioning was obtained by intermittent (10 s/each) or continuous drug infusion during the first 3 min of reperfusion. Left ventricular end-diastolic pressure (LVEDP), left ventricular developed pressure (dLVP), coronary flow (CF), and left ventricular infarct mass (IM) were measured together with the activation status of RISK kinases Akt, p42/44 MAPK and GSK3β.

**Results:**

When compared to hearts subjected to ischemia/reperfusion (iI/R) alone, continuous IRB or LOS administration did not significantly reduce total infarct mass (cIRB or cLOS vs. iI/R, p = 0.2). Similarly, intermittent IRB (iIRB) was not able to enhance cardioprotection. Conversely, intermittent LOS administration (iLOS) significantly ameliorated cardiac recovery (iLOS vs iI/R, p<0.01). Differences between iLOS and iIRB persisted under continuous blockade of AT2R (iLOS+cPD vs. iIRB+cPD, p<0.05). Interestingly, iLOS cardioprotection was lost when BK2R was simultaneously blocked (iLOS+cHOE vs. iI/R, p = 0.6), whereas concurrent administration of iBK and iIRB replicated iLOS effects (iIRB+iBK vs. iLOS, p = 0.7). At the molecular level, iIRB treatment did not significantly activate RISK kinases, whereas both iLOS and iBK treatments were associated with activation of the Akt/GSK3β branch of the RISK pathways (p<0.05 vs. iI/R, for both).

**Conclusions:**

Our results suggest that intermittent losartan is effective in mediating post-conditioning cardioprotection, whereas irbesartan is not. The infarct mass reduction by intermittent losartan seem mainly related on its specific ability to modulate BK2R, and only modestly associated on AT1R blocking properties.

## Introduction

The extent of heart damage is a major prognostic determinant for survival stratification risk in patients undergoing acute myocardial infarction (AMI) [1]. Increased tolerance to a sustained ischemic insult may be achieved by pre-conditioning or post-conditioning strategies, which are supposed to trigger an adaptive response associated with decreased reperfusion-induced arrhythmias, increased recovery of post-ischemic contractile function, and reduced infarct size [2], [3]. Conditioning strategies may exert cardioprotection by facilitating the appropriate time-release of intracellular mediators including catecholamines, adenosine, nitric oxide, and bradykinin (BK) into the coronary circulation [4]. These mediators (either alone or in combination), are thought to potentiate signal transduction cascades involving, among others, phosphatidylinositol-3-kinase (PI3K)/Akt and p42/p44 extracellular mitogen-activated kinases (MAPK1/2), collectively termed the reperfusion injury salvage kinase (RISK) pathway [5]. Signaling downstream the RISK pathway appears to converge on the mitochondria, particularly on the mitochondrial permeability transition pore (mPTP), which is believed to open during the first few minutes of reperfusion in response to mitochondrial calcium overload, burst of oxidative stress, reduced nitric oxide (NO) production, and ATP depletion [6]. Previous studies suggest that early activation of the RISK pathway may confer cardioprotection through the inhibition of mPTP opening [5] via phosphorylation of the glycogen synthase kinase-3β (GSK-3β) with associated modulation of the mitochondrial ATP-sensitive potassium channel (mitoKATP) [7].

Pre-conditioning with ACE-inhibitors and angiotensin type 1 receptor (AT1R) blockers (ARBs) is supposed to enhance cardioprotection by counteracting contractile and mitogenic actions of angiotensin (Ang) [8], preserving BK from degradation and, in some cases, directly activating BK2 receptors (BK2R) [9], [10], [11], [12], [13], [14].

Since pre-conditioning has limited feasibility in AMI setting, much attention is focusing on post-conditioning strategies. Whether cardioprotection from pre-conditioning and post-conditioning use different mechanisms is currently under discussion [15], together with supportive [16], [17] or negative [2] reports. The efficacy of ARBs in post-conditioning strategies has not been deeply explored [18]. On this regard, it is important to consider whether drugs of this class may share similar efficacy. Among ARBs, losartan has an imidazole with Cl and COOH substituents at the carboxylic end of the molecule, while irbesartan has a cyclopentyl ring incorporated in place of the Cl. As a consequence of this different structure, the affinity at AT1R binding site is the lowest for the active form of losartan and the highest for irbesartan [19], [20], [21]. In terms of pharmacokinetic properties, irbesartan is an active drug [22] while losartan is a prodrug whose active metabolites are approximately 4 times as potent as the parent compound [23]. Importantly, losartan exerts a positive modulation on BK levels and activity [24], [25], [26].

When administered in pre-conditioning protocols, losartan-mediated functional recovery and infarct size-lowering ability are partially blocked by the BK2R inhibitor HOE-140 [24], thus suggesting that both BK-dependent and BK-independent mechanisms are important for losartan-mediated cardioprotection. Interestingly, post-ischemic treatment with exogenous BK is able to reduce infarct mass, but improves only partially left ventricular functional recovery [27].

The aim of this study was to evaluate the potential cardioprotective effect of ARBs in post-ischemic administration. Taking advantage of the most cardioprotective post-ischemic algorithm demonstrated for BK [27], we compared the effects of two different ARBs, losartan and irbesartan, under continuous or intermittent administration, and elucidated the signaling pathways activated. Findings obtained suggest that losartan is effective in mediating post-conditioning cardioprotection when administered intermittently, whereas irbesartan is not. Rather than associated to losartan AT1R blocking properties, the infarct mass reduction by intermittent losartan seems mainly related on its specific ability to modulate BK2R and subsequently activate the Akt/GSK3β branch of the RISK pathways.

## Materials and Methods

### Animal experiments

This study was performed in conformance with the Guide for the Care and Use of Laboratory Animals published by the US National Institutes of Health (NIH Publication No. 85-23, revised 1996) and in strict accordance with recommendations in the Guidelines and Authorization for the Use of Laboratory Animals (Italian Government, Ministry of Health). All experimental procedures involving animals were approved by the Committee on the Ethics of Animal Experiments of the University of Bari (protocol number: 60901-X/10; permit number 15/12).

Adult male Sprague-Dawley (SD) rats weighing 250–300 g were obtained from Harlan Italy (Milan) and housed in a temperature-, humidity- and light-controlled room at the animal facility of our Department. Rats were randomly assigned to treatments illustrated under “Experimental protocol”, anesthetized with sodium pentobarbital (80 mg/kg body weight i.p.), heparinized (400 UI/100 g body weight i.p.) and sacrificed by cervical dislocation. All efforts were made to minimize animal suffering.

### Drugs

Losartan potassium was purchased from Santa Cruz Biotechnology; irbesartan, PD123319, HOE-140 and BK were from Sigma. Stock solutions of losartan (10 mM), PD123319 (10 mM), BK (10 mM), HOE-140 (0.2 mM) were prepared in distilled water. Stock solution of irbesartan (10 mM) was prepared in DMSO. Final dilutions were prepared in modified Krebs-Henseleit solution immediately before use. For each drug used, concentration was chosen according to literature data indicating optimal expected effects (see below for specific references). Since drug effects were evaluated on isolated heart, no hepatic metabolic transformation could be expected on administered compounds. Based on both pharmacokinetic and pharmacodynamic properties, losartan was administered at concentration 4.5-fold higher than irbesartan.

### Myocardial function and infarct size in isolated hearts

Hearts from SD rats were isolated and mounted on a Langendorff perfusion system (Radnoti LLC, USA), as previously described [28]. Briefly, excised hearts were immediately subjected to aortic cannulation and perfused with modified Krebs-Henseleit solution continuously gassed with a mixture of 95% O_2_ and 5% CO_2_ (pH 7.4) at 37°C. The perfusion pressure (PP) was kept constant at 80 mmHg in each reservoirs for the whole experimental procedure. Isovolumetric recordings of left ventricular systolic (LVSP) and end-diastolic (LVEDP) pressures were obtained from a balloon catheter inserted into the left ventricle through the auricle (LVEDP set to 5–10 mmHg at the beginning of the stabilization period). Coronary flow was measured by timed collection of the coronary effluent. Left ventricular developed pressure (dLVP) was calculated as dLVP  =  LVSP - LVEDP. After 20 min stabilization, inflow tubing to the hearts was clamped for 30 min to obtain global ischemia. Hearts were then reperfused for 120 minutes. During equilibration, hearts were excluded if they met one of the following criteria: (1) unstable contractile function, (2) coronary flow outside the range of 8–16 ml min^−1^, (3) heart rate below 240 beats min^−1^ or appearance of severe arrhythmia, (4) LVSP outside the range of 60–160 mmHg, once LVEDP was kept constantly between 5–10 mmHg. LVSP, LVEDP, dLVP, and coronary flow were recorded twice before ischemia, and after 5, 15, 30, 45, 60, 90 and 120 minutes of reperfusion.

All data were acquired at a sampling rate of 1 kHz by a 4-channel PowerLab system (ADInstruments, UK) and analyzed using LabChart 7 Pro Software (ADInstruments, UK).

At the end of reperfusion period, infarct size was measured. Briefly, hearts were incubated in freshly prepared 2,3,5 triphenyltetrazolium chloride (TTC 1% w/v phosphate buffer pH 7.4, 37°C, 20 min) and then weighed and frozen (−80°C, 30 min), according to standard methods [29], [30]. Each heart was subsequently sliced in transverse sections (2 mm thick), and each slice weighed and scanned on both sides by a flat-bed scanner (Epson 3490). The infarct area on each color image (TTC unstained) was traced in a blind fashion and measured by planimetry (Image-Tool 2.0 Software NIH, USA). Weight correction of infarct area was performed to avoid that confounding factors such as the thickening of each slice would affect respective infarct area evaluation. Total infarct mass (IM, representing infarct mass with respect to total left ventricular muscle mass) was calculated by summing up for each slice, weight corrected, the extent of infart area according to the following formula: n(*AI/lvA*) x (*Wn/Wtotal*), where *lv*A is the left ventricular infarct area of each slice (n), Wn is the weight of the respective section (n) and Wtotal is the sum of all slice weights [18].

### Experimental protocol

**Figure 1:**
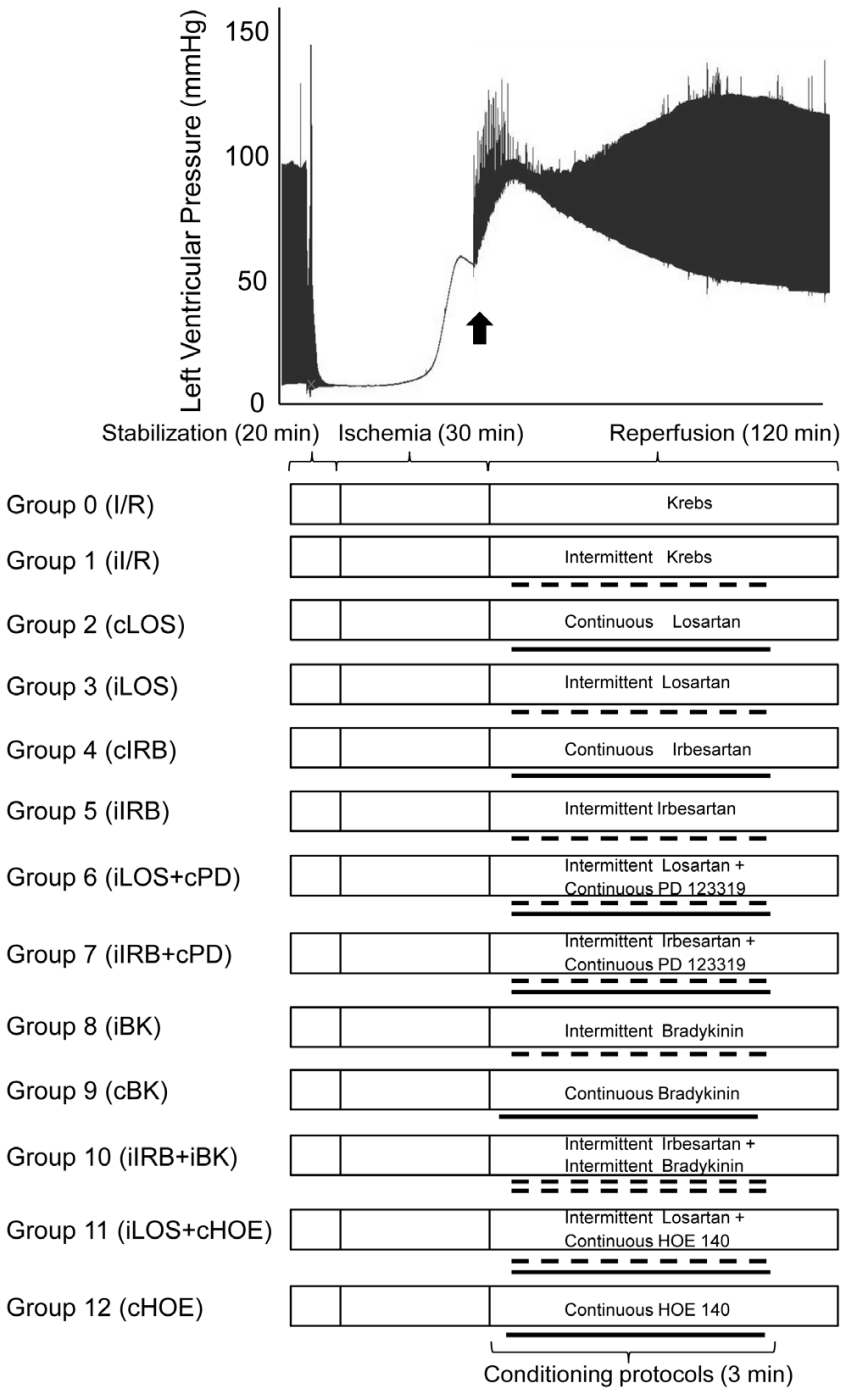
Experimental design. Each heart was isolated and mounted on a Langendorff system. Perfused hearts were stabilized for 20(30 min) followed by reperfusion (120 min). Intermittent post-conditioning algorithms during the initial 3 min of reperfusion alternated 9 cycles (10 sec) of drug infusion to 9 cycles of Krebs' infusion according to protocols illustrated above.

Detailed protocols for post-ischemic treatment are illustrated in Figure 1. Each heart was allowed to stabilize for 20 min. In all groups, after the stabilization period, hearts were subjected to 30 min of global no-flow ischemia followed by 120 min of reperfusion. According to specific protocols, post-ischemic hearts were exposed to intermittent or continuous infusion with different drugs during the first 3 min of the whole reperfusion time, using a second reservoir. For intermittent administration, protocol alternated 9 cycles of 10 s/each with Krebs to 9 cycles of 10 s/each with Krebs alone or Krebs containing the specific drugs. No difference in temperature, oxygen tension or perfusion pressure between reservoirs was measured for the whole experimental procedure.

In Group 0 (I/R, n = 5), hearts were exposed to 30 min of ischemia, followed by 120 min of reperfusion.

In Group 1 (iI/R, n = 8), hearts were intermittently perfused with Krebs (perfusion procedure control).

In Group 2 (cLOS, n = 8), hearts were continuously perfused with losartan (4.5 µM) [31].

In Group 3 (iLOS, n = 9), hearts were intermittently perfused with losartan.

In Group 4 (cIRB, n = 5), hearts were continuously perfused with irbesartan (1 µM) [32], [33]

In Group 5 (iIRB, n = 6), hearts were intermittently perfused with irbesartan.

In Group 6 (iLOS+cPD, n = 6), hearts were intermittently perfused with losartan under continuous administration of the AT2R antagonist PD123319 (1 µM) [32], [33].

In Group 7 (iIRB+cPD, n = 6), hearts were intermittently perfused with irbesartan under continuous administration with the AT2R antagonist PD123319 (1 µM) [32], [33].

In Group 8 (iBK, n = 6), hearts were intermittently perfused with bradykinin (100 nM) [27]

In Group 9 (cBK, n = 3), hearts were continuously perfused with bradykinin (100 nM) [27]

In Group 10 (iIRB+iBK, n = 5), hearts were intermittently co-perfused with irbesartan and bradykinin.

In Group 11 (iLOS+cHOE, n = 6), hearts were intermittently perfused with losartan under continuous administration with the specific BK2R antagonist HOE-140 (1 µM) [34].

In Group 12 (cHOE, n = 4), hearts were continuously perfused with HOE-140 (1 µM) [34].

### Western blot analysis

To evaluate which cytosolic signaling pathway is early activated under each procedure, hearts mounted on the Langendorff perfusion system were stabilized and then subjected to 30 min of global ischemia followed by 10 minutes of reperfusion [35]. Hearts (n = 3 per group) were assigned randomly to treatment groups 1, 3, 5, and 8, as previously described. At the end of reperfusion, samples of the left ventricular tissue were collected, freeze-clamped in liquid nitrogen and then stored at −80°C until further analysis.

Samples were homogenized on ice in cold RIPA lysis buffer containing 1% Nonidet P-40, 0.5% sodium deoxycholate, 0.1% SDS, 50 KIU aprotinin, 100 mM sodium orthovanadate, 10 mg/ml PMSF, and then centrifuged at 4°C for 15 min at 13.000 g. Protein level was determined by Bradford's method [36].

Equal amounts of protein (100 µg) were separated by 10% SDS-PAGE and subjected to immunoblotting with the following primary antibodies (dilution 1∶1000): Akt, p-Akt, p42/44MAPK, p-p42/44MAPK, GSK-3β, p-GSK3β (Cell Signaling Technology, MA). Incubation with HRP-linked antimouse or antirabbit secondary antibodies (Santa Cruz Biotechnology Inc., CA) (1∶3000) was performed for 1 h at room temperature. Immunoblotting results were visualized by Molecular Imager® ChemiDoc™ XRS System (Bio-Rad Laboratories, CA). Images were captured with QuantityOne Software (Bio-Rad Laboratories, CA) and blots quantified by scanning densitometry (ImageJ, NIH, Bethesda, MD).

### Statistical analysis

A power analysis was prospectively conducted to determine the number of rats needed, based on coefficients of variation obtained for similar studies in rodents. A sample size of 5 (in each group) was sufficient to detect 10% differences in infarct area extent (α = 0.05) with a power of 0.80. Results are expressed as mean ± SE of *n* experiments (*n* =  number of rats).

All values were analyzed by a two-way repeated measures ANOVA to determine the main effect of time, group and time by group interaction. If the global tests showed major interactions, a one-factor ANOVA followed by Bonferroni correction analysis was performed between different groups within the same time-point. Statistical differences were considered significant if P value was less than 0.05. All analysis were performed using Statistica Release 7 (Statsoft Institute Inc.)

## Results and Discussion

Post-conditioning holds a promising potential in the clinical setting with respect to pre-conditioning strategies, as no previous information of the ischemic event is required [4]. Physical maneuvers such as repetitive cycles of reperfusion and coronary occlusion (ischemic post-conditioning) are known to enhance myocardial recovery following ischemia [2]. Thus, it was important to preliminary exclude that the switching perfusion procedure employed here was not able to exert any protective effect *per se*. As shown in Table 1, no significant difference was measured in baseline functional parameters from isolated rat hearts subjected to distinct protocols. When hearts subjected to ischemia were intermittently perfused with Krebs/Krebs during the first 3 minutes of reperfusion (iI/R group), we did not observe any significant difference with respect to hearts exposed to classical ischemia/reperfusion (I/R group) (data not shown). Thus, the switching maneuver did not significantly ameliorate ventricular function nor was able to reduce the extension of infarct area *per se*. All results obtained in subsequent experiments were compared to findings of the iI/R group.

**Table 1:** Hemodynamic parameters at baseline.

Groups	Body Weight	Heart Weight	dLVP	LVEDP	Heart Rate	Flow Rate
	(g)	(g)	(mmHg)	(mmHg)	(BPM)	(ml/min)
**I/R**	380±10	1.02±0.1	98±14.3	8±2	265±29	11±5
**iI/R**	378±11	0.98±0.2	101±12.1	9±2	275±31	12±2
**cLOS**	375±12	0.84±0.2	94±8.5	9±1	279±19	11±3
**iLOS**	390±10	0.89±0.2	92±9.9	10±2	279±29	12±3
**cIRB**	392±18	0.95±0.4	90±12	10±1	265±30	11±4
**iIRB**	385±10	0.91±0.1	98±14.1	8±2	269±27	11±2
**iLOS+cPD**	395±12	0.98±0.1	107±12.2	10±1	273±12	10±2
**iIRB+cPD**	400±11	0.96±0.1	103±15.3	10±1	278±32	10±1
**iBK**	370±12	0.97±0.2	102±21.9	11±1	261±19	10±2
**cBK**	389±13	1.01±0.1	99±19.8	9±2	275±17	11±1
**iIRB+iBK**	375±15	0.93±0.1	100±13.9	9±1	276±30	11±1
**iLOS+cHOE**	395±12	0.85±0.1	102±14.1	12±3	263±38	10±2
**cHOE**	392±10	0.94±0.3	98±16.7	10±2	269±22	10±4

Values represent the mean ± SDM for values measured during the last 7 min of stabilization prior to ischemia. *dLVP* developed left ventricular pressure, *LVEDP* left ventricular end-diastolic pressure. No significant difference was found between groups (one-way ANOVA test).

### Intermittent losartan administration reduces infarct mass in hearts subjected to ischemia-reperfusion injury, while irbesartan does not

**Figure 2:**
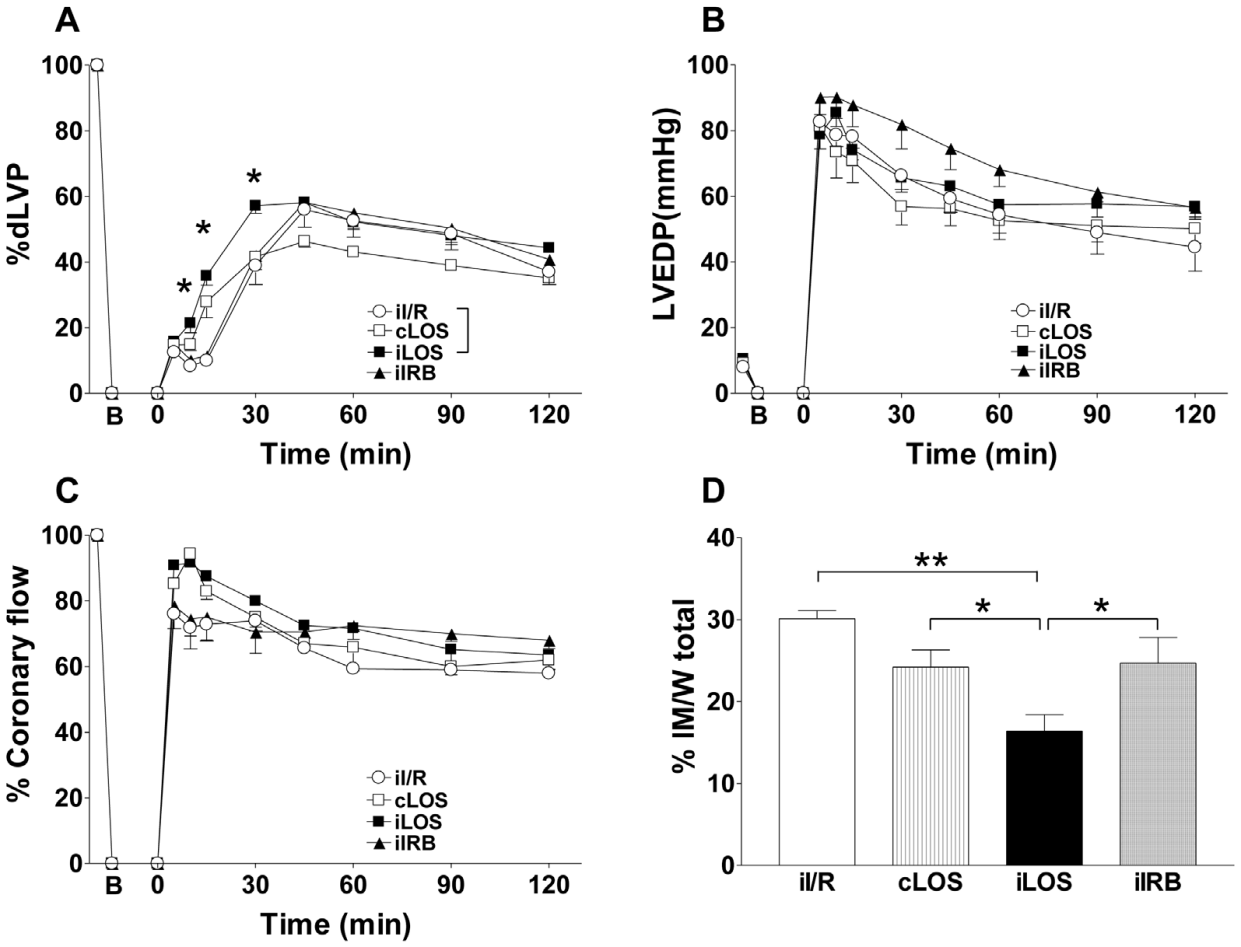
Intermittent losartan post-ischemic administration improves systolic function and reduces infarct mass extent. Hearts exposed to ischemia/reperfusion (iI/R group) were compared to hearts exposed to intermittent (iLOS group) or continuous (cLOS) losartan, as well as intermittent irbesartan (iIRB) administration. In each panel, B corresponds to the beginning of ischemia and time 0 corresponds to the beginning of reperfusion. A. Systolic function. Percent variation of developed left ventricular pressure (dLVP) with respect to baseline level for each group, during the 120 min reperfusion following 30 min global ischemia. B. Diastolic function. Left ventricular end diastolic pressure (LVEDP, mmHg) during the 120 min reperfusion following 30 min of global ischemia. C. Percent variation of coronary flow during the 120 min reperfusion following 30 min of global ischemia. D. Infarct mass. The amount of necrotic tissue is expressed as a percent of the left ventricle mass. Two-way repeated measures ANOVA was employed to determine the main effect of time, group and time by group interaction. A one-factor ANOVA followed by Bonferroni correction analysis was performed between different groups within the same time-point. * p<0.05. ** p<0.01.

When the effects of losartan administered during the first 3 min of reperfusion in intermittent (iLOS group) or continuous (cLOS group) manner were compared (Figure 2), we found that during reperfusion dLVP values were not substantially modified in cLOS group with respect to hearts exposed to switching reperfusion (iI/R group) (panel A). On the other hand, dLVP was significantly higher in iLOS group, when compared to iI/R group, during the first 30 min of reperfusion (p<0.05). Accordingly, during the first 60 min of reperfusion, systolic left ventricular pressure was higher in iLOS group (p<0.01 *vs.* iI/R). Despite higher values of both systolic left ventricular pressure and dLVP in iLOS group during the first hour of reperfusion, no significant differences in both dLVP and LVEDP values (panel B), as well as post-ischemic coronary flow (panel C) were observed between groups at the end of the whole reperfusion time (120 min).

Infarct mass was slightly but not significantly reduced in cLOS group (24.2±2.1) when compared to iI/R group (30.1±1). Conversely, a significant decrease of the infarct mass was observed in iLOS group (16.4±2.0) when compared to either iI/R or cLOS groups (p<0.01; p<0.05, respectively) (panel D). Although apparently protective, early improving in systolic pressure may increase oxygen consumption during critical damage consolidation and thus convert an initial beneficial effect in a pejorative result in the long-term. Consistent with this view, systolic function is not considered an appropriate end-point to study the effects of cardioprotective maneuvers, at least in rodent models [37], [38]. However, in our study, the most protective effects on cardiac recovery were observed under treatments able to ameliorate left ventricular function during the first 30 minutes of reperfusion. Taking into account the limits mentioned above, time course evaluation of left ventricular parameters may help to better understand functional events involved in myocardium recovery during reperfusion.

Results obtained with losartan were then compared with those elicited by irbesartan, the AT1R blocker lacking effects on BK2 receptor. As for continuous losartan, continuous irbesartan administration did not significantly ameliorate post-ischemic cardiac parameters with respect to iI/R group (data not shown). Interestingly, even when administered intermittently, IRB administration was less effective than losartan to improve cardiac recovery (Figure 2, panels A–C), as no significant changes were observed in left ventricular parameters with respect to iI/R group during the first 30 min of reperfusion time. Extent of infarct area in the iIRB grup (24.7±3.1) was comparable to that measured in the cLOS group (p = 0.6), significantly higher than that obtained in the iLOS group (p<0.05), and not significantly reduced vs. iI/R group (p = 0.8) (Figure 2, panel D).

The enhanced cardioprotection obtained in the iLOS group but not in the iIRB group may depend on specific profile of the two drugs, including a different ability to modulate the Ang binding toward the AT2R. Alternatively, since irbesartan is a pure AT1R blocker with no effects on BK signaling while losartan is known to exert a positive modulation on BK2R, losartan cardioprotection might be mediated by a BK-dependent effect. These possibilities were subsequently evaluated with distinct protocols.

### Differences between intermittent losartan and irbesartan post-ischemic administration persist under AT2 receptor blockade

**Figure 3:**
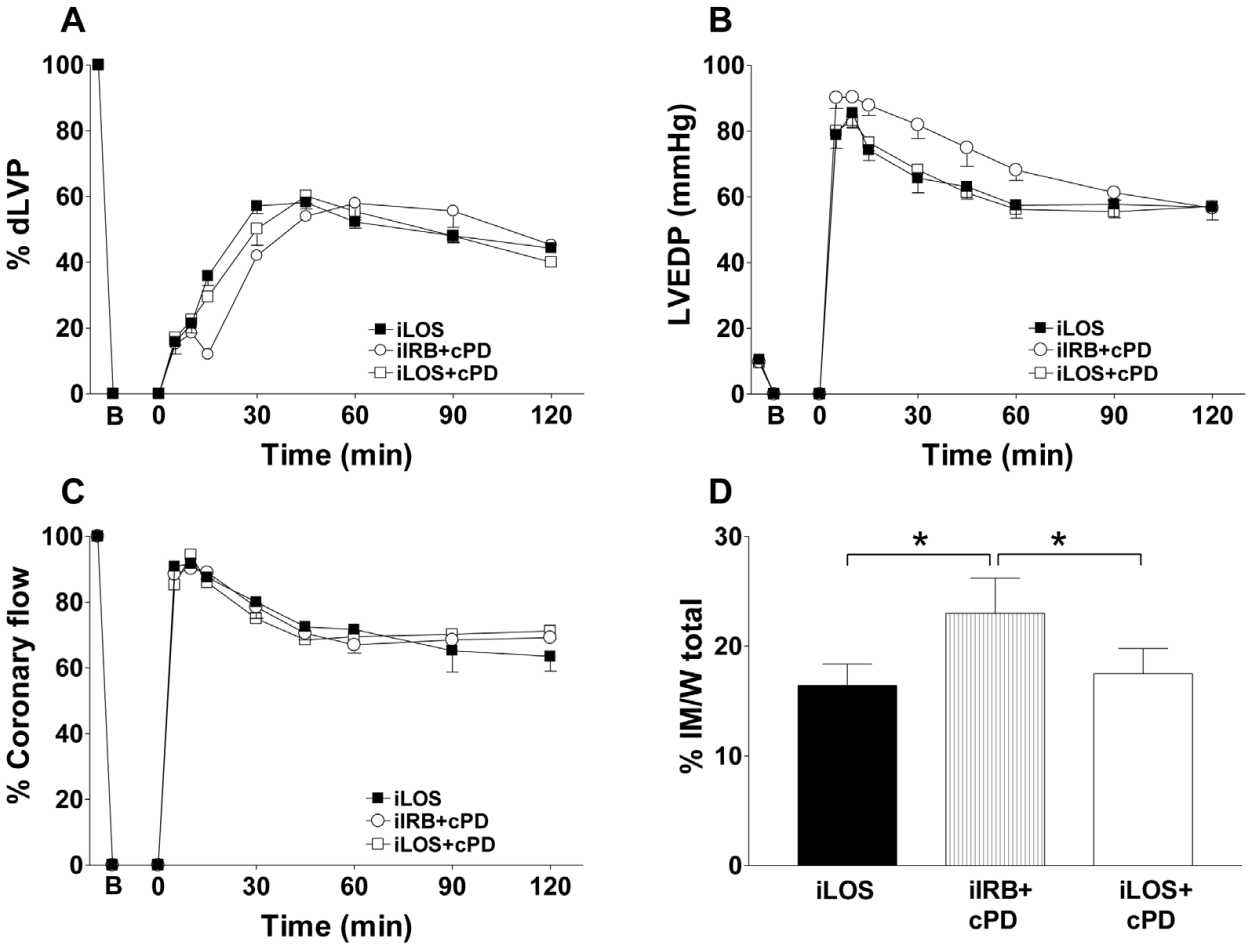
Differences in intermittent losartan and irbesartan post-ischemic cardioprotection do not depend on AT2 receptor blockade. Hearts exposed to intermittent losartan administration (iLOS group), were compared to hearts exposed to intermittent losartan or intermittent irbesartan under continuous continuous AT2 blockade with PD123319 (iLOS+cPD group; iIRB+cPD group, respectively). In each panel, B corresponds to the beginning of ischemia and time 0 corresponds to the beginning of reperfusion. A. Systolic function. Percent variation of developed left ventricular pressure (dLVP) with respect to baseline level for each group, during the 120 min reperfusion following 30 min global ischemia. B. Diastolic function. Left ventricular end diastolic pressure (LVEDP, mmHg) during the 120 min reperfusion following 30 min of global ischemia. C. Percent variation of coronary flow during the 120 min reperfusion following 30 min of global ischemia. D. Infarct mass. The amount of necrotic tissue is expressed as a percent of the left ventricle mass. Two-way repeated measures ANOVA was employed to determine the main effect of time, group and time by group interaction. * p<0.05.

To ascertain whether distinct effects of losartan and irbesartan might depend on modulation of ATR2, results of iLOS and iIRB were compared, respectively, to those obtained in the presence of continuous infusion with PD123319, a pure AT2R blocker (iLOS+cPD; iIRB+cPD). As shown in Figure 3, values of dLVP and LVEDP from hearts exposed to iLOS concomitantly infused with AT2R inhibitor were not statistically different from those obtained in iLOS alone (panel A and B). Likewise, no significant variation was observed for coronary flow levels (panel C), as well as for the extent of infarct mass (panel D) between iLOS+cPD and iLOS groups.

Similarly, values of dLVP, LVEDP, coronary flow and infarct area extent from hearts exposed to iIRB+cPD were not statistically different from those obtained in iIRB group. As a result, infarct area of hearts exposed to iIRB+cPD was still significantly higher than that measured in the iLOS group (Figure 3, panel D: 23±3.2 *vs.* 16.4±2.0; p<0.05) as well as in the iLOS+cPD group (Figure 3, panel D: 23±3.2 *vs.* 17.5±2.3; p<0.05).

These findings suggest that cardioprotection under losartan - but not irbesartan- post-ischemic administration is not dependent on different modulation of Ang system, and further support the hypothesis that losartan activity may be related, at least partially, to BK-dependent effects [25].

### Intermittent losartan post-conditioning depends on modulation of BK system

**Figure 4:**
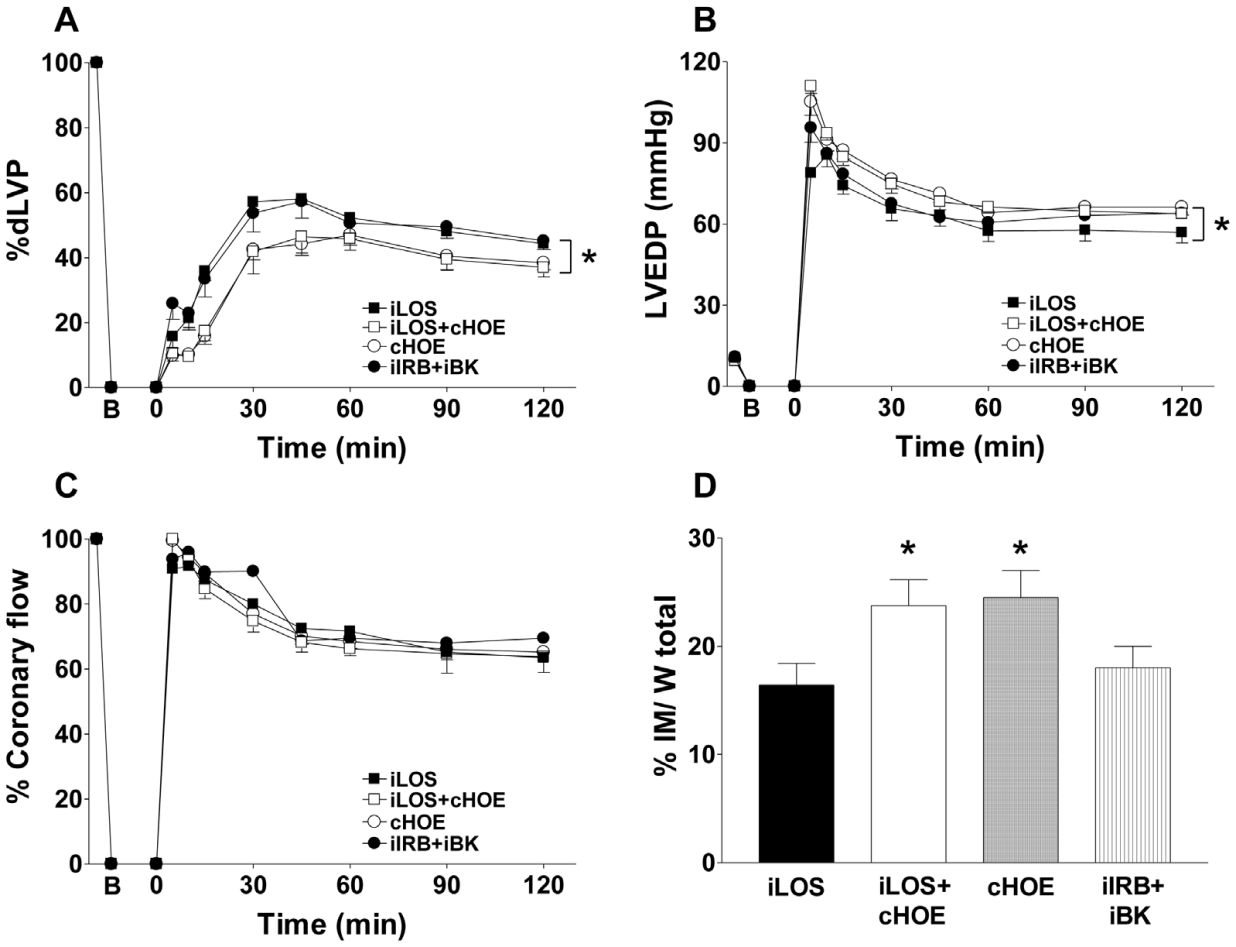
Intermittent losartan post-conditioning is related to modulation of BK system. Hearts exposed to intermittent losartan administration (iLOS group) were compared to hearts exposed to continuous blockade of BK2R with HOE-140 (cHOE group), intermittent losartan under continuous HOE-140 (iLOS+cHOE group) or intermittent administration with both irbesartan and bradykinin (iIRB+iBK group). In each panel, B corresponds to the beginning of ischemia and time 0 corresponds to the beginning of reperfusion. A. Systolic function. Percent variation of developed left ventricular pressure (dLVP) with respect to baseline level for each group, during the 120 min reperfusion following 30 min global ischemia. B. Diastolic function. Left ventricular end diastolic pressure (LVEDP, mmHg) during the 120 min reperfusion following 30 min of global ischemia. C. Percent variation of coronary flow during the 120 min reperfusion following 30 min of global ischemia. D. Infarct mass. The amount of necrotic tissue is expressed as a percent of the left ventricle mass. Two-way repeated measures ANOVA was employed to determine the main effect of time, group and time by group interaction. * p<0.05.

To specifically evaluate whether cardioprotection obtained in iLOS group would depend on intermittent activation of BK2R by losartan itself, results from iLOS group were compared to those obtained in hearts simultaneously and continuously infused with HOE-140, an antagonist of BK2R (iLOS+cHOE group) (Figure 4). Under this condition, the blocking effects of losartan on AT1R were maintained, but its putative effects on BK2R were completely abolished. Of all functional parameters examined, dLVP values and LVEDP values were significantly worsened in iLOS+cHOE group with respect to iLOS group (panel A, p<0.05; panel B, p<0.01, respectively).

Interestingly, continuous perfusion with HOE (cHOE group) did not further worsen cardiac recovery or increase infarct area with respect to I/R group (24.5±2.5 *vs.* 30.1±1.0; p = 0.09). This helps to rule out any direct, deleterious effect of HOE in post-ischemic recovery. As expected, deterioration of cardiac performance in iLOS+cHOE group was accompanied by a significant increase of infarct mass *vs*. iLOS group (panel D: 23.7±2.4 *vs.* 16.4±2.0; p<0.05), with an infarct area extent not significantly different from that in the iI/R group. This further supports the hypothesis that cardioprotection obtained by intermittent losartan is related to its ability to enhance endogenous BK activity. Consistent with this, functional parameters as well as infarct mass extent obtained under intermittent irbesartan co-infused with exogenous BK (iIRB+iBK group) were comparable with those obtained in the iLOS group (Figure 4, panels A–D). Thus, co-administration of BK and irbesartan mimicked the infarct-sparing effect provided by intermittent losartan alone.

**Figure 5:**
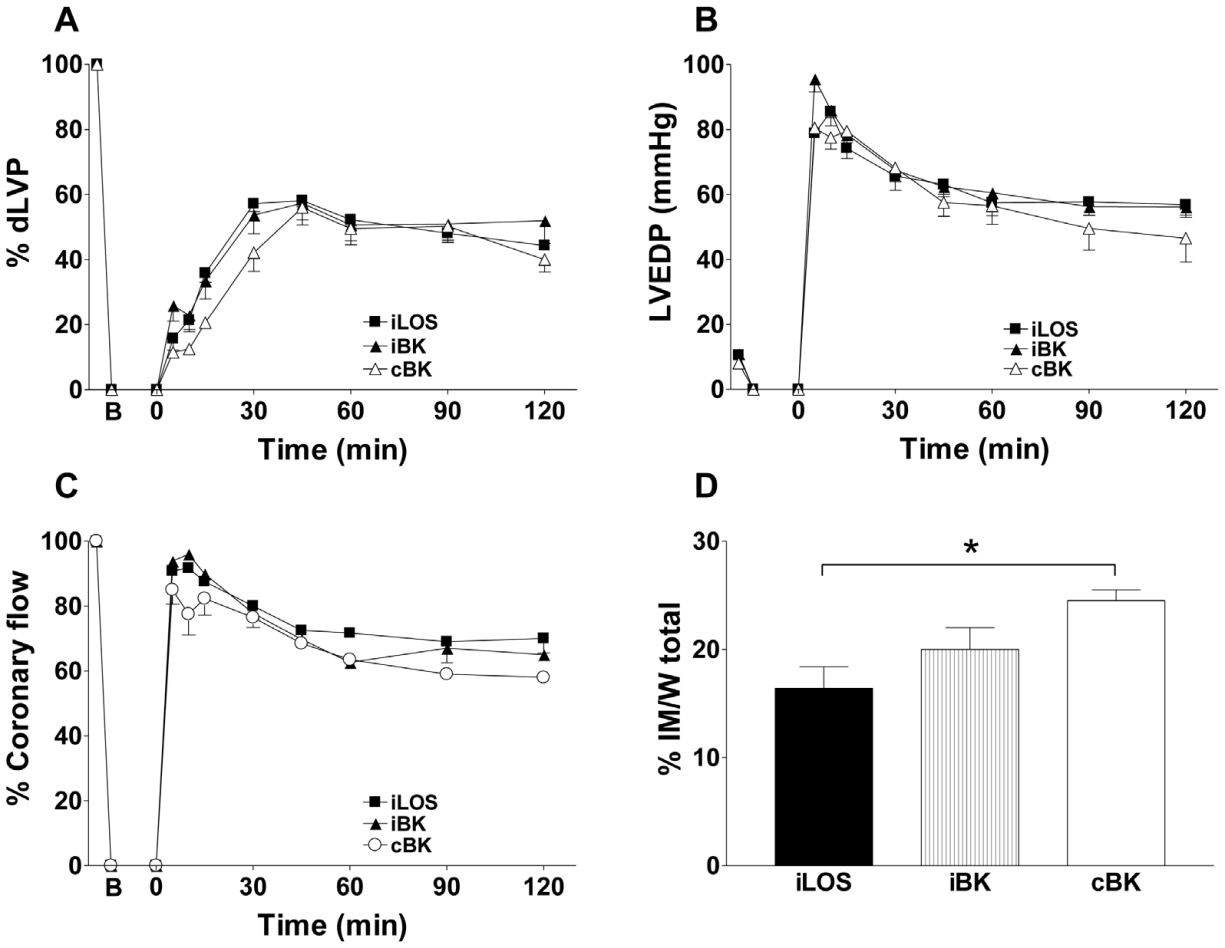
Intermittent losartan or BK administration exert comparable post-ischemic cardioprotection. Hearts exposed to intermittent losartan (iLOS group) were compared to hearts exposed to intermittent (iBK) or continuous (cBK) BK administration. In each panel, B corresponds to the beginning of ischemia and time 0 corresponds to the beginning of reperfusion. **A**. Systolic function. Percent variation of developed left ventricular pressure (dLVP) with respect to baseline level for each group, during the 120 min reperfusion following 30 min global ischemia. **B.** Diastolic function. Left ventricular end diastolic pressure (LVEDP, mmHg) during the 120 min reperfusion following 30 min of global ischemia. **C.** Percent variation of coronary flow during the 120 min reperfusion following 30 min of global ischemia. **D.** Infarct mass. The amount of necrotic tissue is expressed as a percent of the left ventricle mass Two-way repeated measures ANOVA was employed to determine the main effect of time, group and time by group interaction * p<0.05.

As additional confirmation, the extent of losartan-mediated cardioprotection from iLOS group was further compared to that obtained in post-ischemic hearts infused with exogenous BK, either intermittently (iBK group) or continuously (cBK group) administered (Figure 5). Consistent with literature data, iBK substantially reduced infarct area with respect to iI/R group (20.0±2.0 *vs.* 30.1±1.0, p<0.01), although left ventricular functional parameters were not substantially ameliorated. As expected, values of dLVP (panel A), LVEDP (panel B), coronary flow (panel C) and infarct mass extent (panel D) in iBK group were not statistically different from those in iLOS group. Finally, when compared to iLOS group, continuous BK administration did not significantly reduce infarct area extent as efficiently as iBK (Figure 5, 24.5±1.0 *vs.* 16.4±2.0, p<0.05).

Thus, in line with previous studies evaluating the efficacy of BK in post-conditioning [27], post-ischemic cardioprotection exerted by losartan may be ascribed mainly to its ability to activate the BK2R [18], [34].

As for mechanisms involved, recent evidence suggests that BK2R activation in response to hemodynamic shear stress in endothelial cells [39], and by mechanical or ligand-dependent stimulation in the heart, may favor the assembly of a caveolar signaling platform (signalosome). This is the first step in a cascade of intracellular events culminating with inhibition of the mPTP and limitation of apoptotic processes [40]. It is known that, under ischemia, kallidin-like peptides such as Arg-kallidin are produced [9], [41]: these peptides may also bind to BK2R with subsequent formation of reactive oxygen species (ROS) by a NO/cGMP-mediated mechanism. In turn, ROS act on mitoKATP channel to prevent the opening of mPTP [42]. Thus, besides BK, a variety of mediators may modulate BK2R activity. This supports the hypothesis that losartan may favor the recruitment of a substantial number of BK2R undergoing conformational changes, with subsequent increased signalosome formation and signaling activation.

Effective cardioprotection can be observed only when losartan is administered intermittently: this modality resembles effects observed under transient episodes of ischemia/reperfusion (ischemic post-conditioning), where cardioprotection is achieve by short-term, repeated cycles of coronary occlusion/reperfusion [2], [16].

On this regard, it is interesting to observe that continuous activation of the BK system (cBK group) does not confer additional protection while continuous blockade of the BK2R (cHOE group) does not further worsen cardiac recovery. Altogether, these findings reinforce the concept that is the appropriate triggering of cellular pathways and the subsequent correct availability of myocardial substrates that plays a major role on conditioning strategies [43]. When modulated in a pulsatile (intermittent) manner, the BK system is a major determinant for post-conditioning protection. Consistently, the maximal protective effects of losartan are not achieved under continuous infusion, but only when the drug is intermittently administered.

### Activation of RISK kinases under post-conditioning treatments

**Figure 6:**
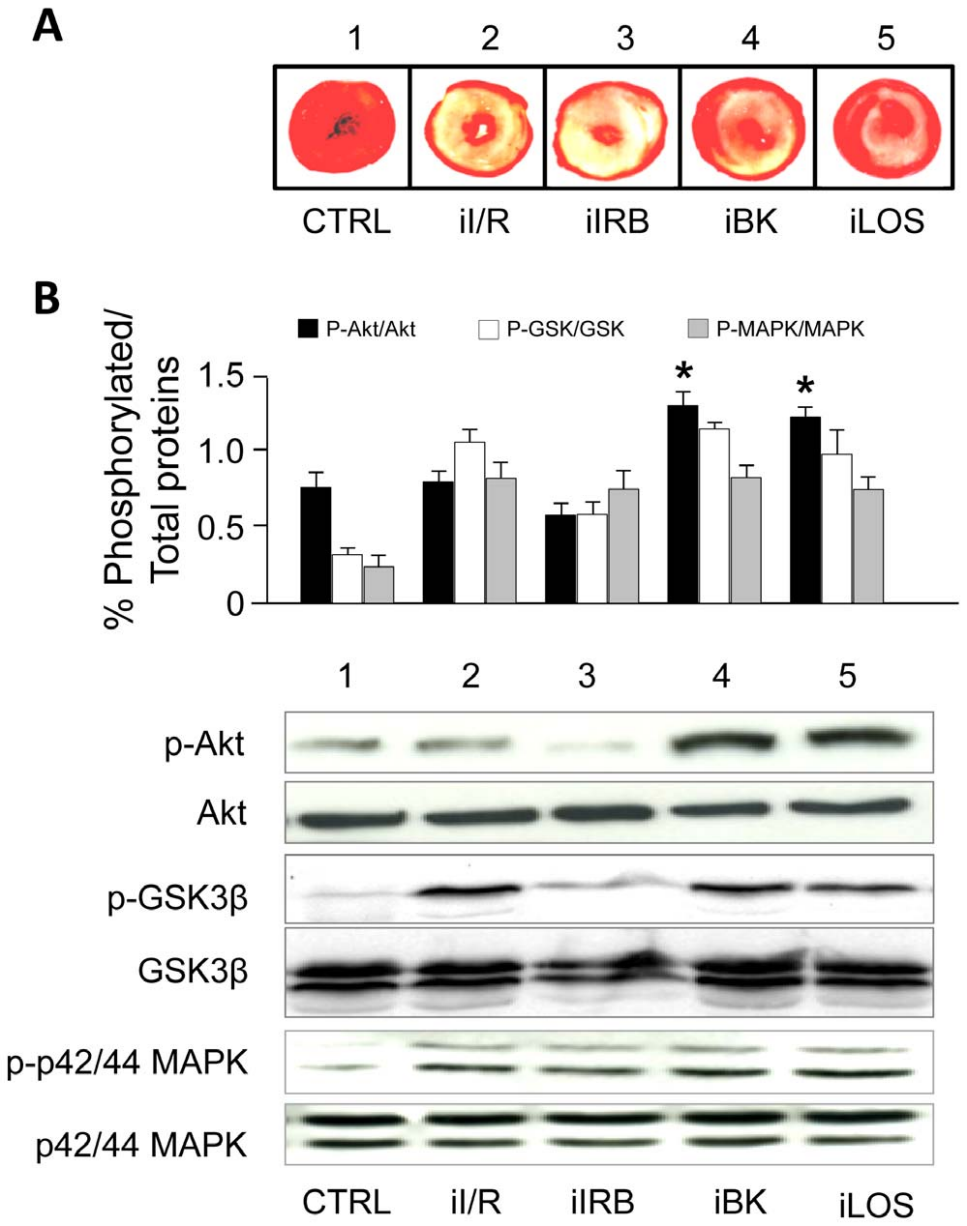
Activation of RISK pathway kinases under post-ischemic intermittent treatments. **A**. Hearts were cut into transverse slices and stained with TTC to differentiate necrotic (unstained) from viable (red) myocardium. Representative images of each treatment are visualized. **B**. Hearts were homogenized and lysates immunoblotted for phosphorylated and total isoforms of the indicated proteins. For each experimental condition, the ratios of phosphorylated/total-Akt, phosphorylated/total GSK-3β and phosphorylated/total p42-44MAPK expressed as the mean ± SEM of at least 3 independent experiments (each run in triplicate) are shown in the upper panel, and representative immunoblots are shown in the lower panel. A one-factor ANOVA followed by Bonferroni correction analysis was performed.* p<0.05 *vs*. iI/R groups. *CTRL, non ischemic hearts; iI/R, hearts exposed to ischemia followed by intermittent reperfusion with Krebs; iIRB, hearts intermittently perfused with irbesartan; iBK, hearts intermittently perfused with exogenous BK; iLOS, hearts intermittently perfused with losartan*

Figure 6 (panel A) shows the typical extent of infarct area for treatments indicated. To evaluate whether reduction in heart infarct from the iLOS and iBK groups (but not in the iIRB group) might be related to specific activation of RISK signaling pathway [35], we examined the phosphorylation states of Akt (ser473), p42/44 MAPK (Thr202/Tyr204) and GSK3β (ser9) in hearts exposed to our post-ischemic treatments (Figure 6, panel B). We found that in the iIRB group the p-Akt/Akt ratio and the p-GSK3β/GSK3β ratio were slightly (although not significantly) reduced, while no difference was found in the p-MAPK/MAPK ratio with respect to the iI/R group. Conversely, in both the iBK and iLOS groups, the p-Akt/Akt ratio was significantly increased (p<0.05), although both the p-GSK3β/GSK3β and the p-MAPK/MAPK ratios were unaltered with respect to values measured in the iI/R group.

The importance of GSK3β inhibition (by phosphorylation at serine 9) in cardioprotection is supported by several studies [29], [44]. However, recent evidence suggests that modulation of GSK3β alone is not sufficient to elicit pre- and post-conditioning: for example, transgenic mice with an inactive mutant of GSK3β have shown either abrogation [45] or preservation [46] of post-conditioning protection on infarct size. In part, this may be explained by the fact that small amount of ROS generated by ischemic-mediated mitoKATP opening are able to increase GSK3β phosphorylation acutely [47], but that a cardioprotective effect can be obtained only when GSK3β inhibition is sustained. There is a broad consensus that the first step in cardioprotective signaling is the activation of cytosolic pathways, followed by the translocation/phosphorylation of RISK effectors to the mitochondria; from here, the activated signaling rebounds to cytosolic elements whose recruitment is required to achieve the cardioprotective result [48]. The phosphorylation/activation of cytosolic Akt is considered a pivotal event [49]. In line with this, phosphorylation/activation of cytosolic RISK kinases in our study was evaluated at the earliest step of reperfusion (10 min) with respect to infarct size assessment (120 min). A significant increase in Akt phosphorylation was detected in hearts subjected to iLOS and iBK treatment, whose infarct mass was smaller with respect to that measured in iI/R or iIRB groups. At this time of the study, we observed similar levels of the phosphorylated GSK3β form among iI/R, iLOS and iBK groups. As mentioned before, it is possible that activation of PI3K/Akt in response to either iLOS or iBK – but not iIRB- contributes to maintain optimal phosphorylation of GSK3β, and therefore explain the higher cardioprotective effect obtained under these two conditions.

Since the mPTP formation is one of the major regulators of cardiac injury, and inhibition of mPTP is widely recognized as the end-effector of cardioprotection [50], it is very likely that cardioprotective effects of intermittent losartan may depend on inhibition of mPTP opening. In this sense, the qualitatively similar activation of RISK signaling pathway by both iBK and iLOS support the idea that iLOS, as iBK, involves downstream cellular events converging on modulation of mPTP. In isolated rabbit hearts [51] or mouse hearts [52], post-conditioning has been found associated to activation of the p42/44 MAPK and not the Akt pathway. We did not observe any significant difference in phosphorylated p42/44 MAPK levels among groups. Whether p42/44 MAPK phosphorylation remains constant throughout the whole reperfusion time or follows a specific pattern remains to be elucidated. Nevertheless, these findings further support the concept that, more than the singular role of Akt, MAPK and GSK3β, is the complex integrated signaling responsible for the protective effect of the RISK pathway.

Although the inhibition of mPTP is not directly demonstrated, and a causal relationship between activation of a specific signaling and the resulting biological effects cannot be directly assumed under our experimental conditions, the observation that components of the RISK signaling are differentially activated under irbesartan or losartan post-ischemic administration suggests that RISK pathway may significantly contribute to overall protection in losartan-mediated post-conditioning.

## Conclusions

This study provides the first evidence that losartan, when administered intermittently in the early reperfusion period, is effective in post-conditioning cardioprotection; conversely, irbesartan does not seem to reduce infarct size, either under continuous or intermittent administration. The infarct mass reduction by intermittent losartan seem mainly related on its peculiar ability to modulate the BK2R, since iLOS cardioprotection was lost under continuous blockade of BK2R, and mimicked by simultaneous intermittent administration of irbesartan and BK. In addition, functional and morphological outcomes of irbesartan and losartan were associated with differential activation of cytosolic RISK pathway kinases. Findings illustrated here may contribute to discern which class component, among ARBs, may be more appropriate for post-ischemic cardioprotection.
